# Structural Problems Demand Structural Solutions: Addressing Domestic and Family Violence

**DOI:** 10.1177/10778012231179212

**Published:** 2023-06-07

**Authors:** Evelyn Rose, Charlotte Mertens, Jennifer Balint

**Affiliations:** School of Social and Political Sciences, University of Melbourne, Parkville, Australia

**Keywords:** domestic and family violence (DFV), sexual and gender-based violence (SGBV), violence prevention, structural justice, community legal centers

## Abstract

Despite the global recognition of domestic and family violence (DFV) as an outcome of unequal power relations between men and women, dominant frameworks for addressing DFV do not target the structural nature of the problem. Drawing on research conducted in partnership with the Federation of Community Legal Centres in Australia, we argue that a distinction needs to be made between what is genuinely structural change and what is system reform. Using intersectional feminist and decolonial theory and praxis, we reflect on what a structural approach to DFV could look like: one that confronts and actively tries to change the structural conditions that give rise to women's individual and collective vulnerability and victimization.

## Situating the Structural Nature of DFV

Domestic and family violence (DFV)^1^ is an urgent global problem. It is the most common form of sexual or gender-based violence (SGBV), with around a third of women worldwide having experienced victimization (NCADV, 2020; UNODC, 2019). Since the onset of the COVID-19 pandemic, DFV has increased and diversified at unprecedented rates in all societies (Boserup et al., 2020; Boxall et al., 2020). Although the problem had already been widely described as an “epidemic” and a “pandemic” (e.g., Moreira & Pinto da Costa, 2020), the Executive Director of United Nations Women recently described violence against women and girls as the “shadow pandemic” of the COVID-19 era (Mlambo-Ngcuka, 2020).

DFV is widely recognized across the political, policy, and academic spheres as a form of SGBV with multiple gendered dimensions. It is gendered in the sense that most cases involve male perpetrators and female victims. Worldwide, this dynamic characterizes most cases, and an even higher percentage of those involving medical or legal intervention (Bradel et al., 2019; Flood, 2012; Kimmel, 2013). In particular, repetitive, threatening, escalating, and dangerous abuse that is more aptly described by terms such as coercive control, intimate terrorism, or domestic torture (Graham, 1994; Johnson, 1995; Kelly, 1988; Pain, 2012; Stark, 2007) is overwhelmingly perpetrated by men against women.

Across the globe, there are a diversity of DFV responses in place ranging from targeted offender accountability and rehabilitation programs, victim safety apps and risk assessment tools, and educative prevention campaigns, to specialist law enforcement units and courts. Within this diverse response repertoire, there is widespread consensus that responses need to be survivor-centered and maintain the principles of the early feminist movement. These principles acknowledge that each individual woman is best placed to understand her circumstances, risks, challenges, and needs (Dutton et al., 2015; Goodman & Epstein, 2008). They also recognize DFV as a multifaceted problem that diffusely impacts victims in a range of ways and not only threatens their physical safety but also their psychological, emotional, and spiritual health; social, community, and professional connections; and financial safety and security. In fact, scholars increasingly document the many ways that DFV causes everyday insecurity for women that lasts well beyond immediate victimization (Voth Schrag et al., 2019; Walklate et al., 2019). These insights inform the need to provide support for victim-survivors across extended time periods, thereby challenging the mistaken notion that victims only experience “crisis” and need assistance when attempting to leave an abusive partner. Indeed, scholars and policy reports have shown that in order to facilitate best practice responses to DFV, the notion of “crisis” must be conceptualized very broadly and with an understanding that crises are often a drawn-out succession of events over years, decades, and sometimes even survivors’ lifetimes (Cameron, 2014; Guggisberg, 2018; Laing, 2017).

Yet, with DFV continuing at epidemic rates without signs of abatement, we must ask the uncomfortable question “do current approaches sufficiently address the problem of DFV?.” In this article, we critically examine contemporary responses to DFV and argue that although many initiatives are worthwhile and important, a distinction needs to be made between what is genuinely systemic and structural and what is merely a change or reform to existing responses. Our concern is that, although progressive structural analyses of DFV have been around for many decades, there has been limited translation of these into concrete structural prevention strategies. Indeed, as far back as 1993, official international policy documents explicitly named DFV as an outcome of women's subordination and their symbolic and substantive disadvantage at all levels of society (UNGA, 1993). For instance, the 1993 Declaration on the Elimination of Violence against Women recognizes that:

violence against women is a manifestation of *historically unequal power relations* between men and women, which have led to domination over and discrimination against women by men and to the prevention of the full advancement of women, and that violence against women is one of the crucial social mechanisms by which women are forced into a subordinate position compared with men. [authors’ emphasis]

Indeed, feminist scholars have comprehensively conceptualized how DFV and other SGBV are caused by sexual and gender inequality (Kelly, 1988; MacKinnon, 1994), and it is for this reason that it has been understood as a form of *structural violence* (Scheper-Hughes & Bourgois, 2004). Inequalities are structured in societies, they are historically and transnationally produced, are legitimated over time, and reproduced in people's everyday realities (Balint et al., 2020; Galtung, 1969). DFV, like all forms of SGBV, is coproduced by transnational structural forces such as capitalism, neoliberalism, and colonialism, all of which are informed by a patriarchal gender order (see Federici, 2004; Tamale, 2020). DFV, occurring within the state-sanctioned patriarchal institution of the family, is thus key for analyzing the intersection of structural forms of violence and the multiple actors involved (nation-states, public and private institutions, and individual perpetrators).

In many contemporary contexts, sexual and gendered inequality is frequently—and appropriately—cited as the “underlying cause” or “root” of DFV. For example, in our local context of Victoria, Australia, the landmark 2016 Royal Commission into Family Violence (RCFV) identified in its report that DFV is “deeply rooted in power imbalances” and has causes that “include gender inequality” (RCFV, 2016, p. 2). From this progressive structural framing, one might expect a prevention approach and set of initiatives that target substantive sexual and gendered inequality. However, as we critically consider in this article, responses have tended not to pursue a structural approach. This is because once sexual and gendered inequalities are rearticulated at the local level as DFV in households, families, and communities, the problem becomes localized (and individualized) while historical, national, global, and structural dynamics are invisibilized. There is thus widespread inconsistency between the conceptualization of DFV, its causes, and preventative and remedial responses. This is not to argue that current approaches have no value. Yet we problematize many initiatives’ claims to tackle DFV “at its root,” and the way they are framed as “holistic” and “systemic” responses that address the problems underlying the harm. Indeed, when critically unpacked, initiatives are broadly exposed as maintaining a focus on problematic individuals and attitudes rather than problematic histories, institutions, and structures.

In the next section, we critically analyze and categorize some dominant contemporary responses in our local context, identifying how some best practice principles (such as survivor-centered and long-term approaches) are being engaged through valuable initiatives, but also critically considering the extent to which these can be understood as structural. In the final section of the article, we engage in a broader critical feminist conversation that considers what a structural response to DFV could look like: one that confronts and actively tries to change the structural conditions that give rise to women's individual and collective victimization. Using examples of some programs by Community Legal Centres (CLCs) in Australia, we begin to trace the contours of a more concerted structural approach to DFV prevention. We complexify the notion of the perpetrator to consider a broader field of culpability, further consider what it means to conceptualize this violence *as* structural, and tackle the difficult issue of what it might mean to address DFV as a structural problem, the inherent challenges within this frame, and possible ways forward. Amid more targeted prevention and redress efforts, we emphasize the importance of striving for genuinely structural solutions to DFV and indeed all forms of sexual, gendered, and other structural harms.

## Categorizing and Critically Considering Contemporary DFV Approaches

With the political recognition of the severity and urgency of addressing DFV, huge funding opportunities for anti-DFV initiatives and a vast plethora of responses have been developed. Yet despite the widespread acknowledgment that DFV stems from structural sexual and gender-based inequality, contemporary responses tend not to pursue a structural approach to the problem. Our local context of Victoria, Australia, exemplifies a progressive approach to DFV which is reflected in many contexts across the Global North. The Victorian State Government initiated a Royal Commission into Family Violence (2016) that heard from survivors and practitioners, amongst others, with the result being a political commitment to implement every single recommendation in the Commission's final report. Further developing our previous analyses (Rose et al., 2018), we argue that responses can be broadly understood within three categories: individualized approaches, attitudinal cultural change, and systems reform. Our concern is that, while these are all important elements within a DFV response toolkit and they can *together contribute to* structural change over time, they do not necessarily align with a structural framing of the problem, nor do they adequately address its structural dimensions.

### Individualized Approaches

DFV is commonly conceptualized and addressed as an individual, interpersonal problem. The account produced by RCFV (2016), for example, focuses heavily on individual perpetrators, depicting them as ill, addicted, poorly educated, or defectively acculturated men who require treatment, education, condemnation, or punishment (Rose, 2022; Yates, 2020). This flows through to perpetrator-focused responses that compel offenders to take responsibility for their harmful attitudes and behaviors. In addition to criminal and civil legal sanctions, this is often through Men's Behaviour Change Programs. Although there is value in such programs, they also require ongoing critical interrogation. This is because they can promote unhelpful and inaccurate assumptions about DFV at the same time as having a variable and limited concrete impact on recidivism (Babcock et al., 2016; Cantos et al., 2019; McGinn et al., 2016). Firstly, these programs often adopt an individual and microsituational approach to DFV prevention: for example, through working on an individual man's ability to control or suppress anger and substitute nonharmful behaviors for violent behaviors.

Notably, the anger management aspects of these programs can conflate DFV with physical assault, which does not reflect the complex and pervasive reality of the harm. Regimes of coercive control are not characterized by random incidents such as outbursts of anger and assault but rather a constellation of controlling, intimidating, and threatening tactics which are deliberately and consistently administered, not only when the offender is angry (DeKeseredy, 2016; Flood, 2012; Johnson, 2011; Kimmel, 2013; Meyer & Frost, 2019; Stark, 2007). Although non-physical forms of violence such as emotional and economic abuse are increasingly recognized in official criminal and civil codes across common law contexts,^2^ survivors continue to experience difficulties having these less visible and direct forms of violence recognized and responded to. Despite widespread evidence to the contrary, there is a persistent perception that invisible abuse is less serious or harmful than injurious physical or sexual violence (Cameron, 2014; McKenzie et al., 2016; Murray & Powell, 2009). The physical assault focus of many perpetrator programs misses this. Moreover, Westmarland et al. (2010) caution against measuring the “success” of such programs via traditional outputs such as the number of people commencing or completing programs or by measuring fewer violent “incidents”; rather, they argue that success needs to account for diverse and complex forms of abuse and stakeholder needs.

Another facet of the individualized approach to addressing DFV is a focus on victim-survivors through what we term a medicalized approach. This occurs when DFV or other forms of SGBV are treated as the victim's medical or health issue, which in practice means that services/programs primarily address DFV by referring victims to health professionals. This generally means that the success (or otherwise) of these programs is measured quantitatively: that is, according to the number of referrals rather than through qualitative experiential impacts for victims. This is reflected in the literature that analyzes individualized responses to victim support that target specific medical needs (Burton & Carlyle, 2021; Downing et al., 2021). Although a necessary part of a multifaceted approach, and often critical for women's individual health needs, a medicalized approach can again individualize DFV.

A medicalized approach to DFV can also pathologize the victimized individual woman, whose body becomes the problem to be remedied, and from whom an agentic male perpetrator, a harmful family environment, or an oppressive patriarchal society, are far removed. It also facilitates the avoidance of deeper and structural inequalities—intersecting sex, race, and class-based histories and persistent oppressions—which are widely recognized to underpin DFV and all forms of VAW. As Ticktin (2011, p. 255) argues in the context of conflict-related sexual violence,

medicalisation transforms gender-based violence into an emergency illness requiring immediate intervention … it narrows gender-based violence … [to something] done to specific parts of the biological body, which are then treated by biomedicine; [it removes] … the fuzziness of context and culture.

Because dominant medico-legal narratives generally focus on singular dimensions and identities of individuals, and because sexual and gender inequalities are inscribed into these hegemonic structures themselves, the question must be raised if the structural nature of DFV can be addressed through these institutions. Having DFV services directed and operated by medical and legal disciplines, two dominant power structures in Western liberal societies which are themselves implicated in the production and reproduction of DFV (Rose, 2015, 2022), is a problematic conundrum. It means that services are limited by the inherent frameworks and confines of their disciplinary roots and demands that we pay careful attention to what they can and cannot offer.

### Attitudinal Cultural Change

The second element that dominates responses in our local and comparable contexts is attitudinal cultural change. This is primarily pursued through education programs at the individual, school, or community level and which focus on promoting attitude and behavior change. School and community organization-based programs such as the Respectful Relationships program by the Victorian Government educates about equality in intimate relationships and effective and nonviolent interpersonal communication (VicGov, 2017). These are undoubtedly valuable initiatives. Yet this model of DFV prevention through cultural change focuses on individuals and attitudes rather than institutions and substantive structures. Claims that such initiatives address inequality are inaccurate, as the model equates inequality with “community attitudes” (RCFV, 2016, p. 2). The focus of these initiatives on micro-level change does not target the inequality “root” of DFV but rather the *symptoms* of inequality.

It is not just restrictive gender roles, discriminatory beliefs, or harmful individual and community attitudes that perpetuate violence against women—it is also substantive structural inequality (CIJ, 2015). While violence-supportive norms may be *closely linked* to sexual and gendered structural inequality, they are not the same thing; it is equally necessary to take substantive collective action to change the underlying determinants of violence against women (Salter, 2016). This means that change is needed to the way that society is structured and how it functions to reproduce material conditions that subordinate and discriminate against women. We will consider this further in the next section.

An individualized and cultural approach to DFV is by no means unique to Western nations or indeed to SGBV in the family. Similar patterns are identified in international anti-SGBV campaigns and interventions (see Mertens & Myrttinen, 2019; Mertens & Pardy, 2017; Salter, 2016), where violence tends to be attributed to problematic individual attitudes and behaviors rather than substantive, institutional, structural, and state-level policies and practices. Prevention initiatives are largely disconnected from material political, economic, and social conditions, and instead focus on providing immediate, proximate therapeutic services to victims, or on challenging the violence-supportive attitudes and problematic masculine behaviors of specific men or male collectives. Shifting measures away from the structural and toward the individual and attitudinal enables a set of inherently more palatable solutions than society-wide efforts to radically interrogate, dismantle, or reassemble societal structures and institutions (Salter, 2016). Although valuable at the individual level, such approaches obscure the deeper institutional and structural dimensions of DFV as a symptom and a tool of sexual and gendered inequality.

### Systems Reform

The third element within many mainstream DFV approaches is systems reform. Recognizing local, national, and global exposure of the widespread “failings of the [domestic and] family violence system” (see Goodmark, 2018; Groves & Thomas, 2014; Meyer & Frost, 2019; RCFV, 2016, p. 9), there has been a strong focus on better connecting the legal, medical, and social service systems that respond to DFV, and creating more effective and streamlined ways for survivors to access existing services. For example, there have been efforts to improve information-sharing across government agencies, reduce the replication and overlap of services, mandate DFV awareness education for first responders and other system workers, create new risk assessment and reporting tools, develop specialist law enforcement units and courts, and introduce official recognition of the value of lived experience expertise into the DFV workforce (Rose et al., 2018). A key body of work reflects the systems reform trend, for instance, promoting or evaluating various tools and interventions for enhancing DFV detection, risk assessment, and service response (Babcock et al., 2016; Hegarty et al., 2015).

An important element within systems reform—and considered international best practice—is service integration. In Victorian, other Australian, and comparable overseas jurisdictions, lack of service integration has been recognized as a major inhibitor to both individual help-seeking efforts and broader violence prevention (European Institute for Gender Equality, 2015; RCFV, 2016; VicHealth, 2014). The RCFV's Report (2016), for example, identifies the problem of “siloing”: where specific services and elements of services needed by a DFV victim at various times are administered in an isolated and dislocated fashion. Siloing is problematic because lack of integration, coordination, and poor communication have been identified as key deficiencies in local and other DFV service systems. The fragmentation of services often leads to “referral fatigue,” where the likelihood of people successfully acting upon a referral decreases each time they are referred to another representative within an agency, or to another agency, service, or program (Pleasence et al., 2014). These issues underscore the need for approaches that unify, simplify, and coordinate services across the sector and make service navigation easier, particularly for the most vulnerable people with complex, intersecting needs (Campo et al., 2014; Hoyle & Palmer, 2014). It is part of recognizing that DFV victims face a broad set of related legal, social, economic, and health problems which also intersect with experiences of everyday adversity. This knowledge has propelled efforts to streamline, connect, and combine DFV-related services to make them more accessible and user-friendly for victim-survivors (see Ross et al., 2016).

Building on the need for integrated and colocated services for DFV is the “one-stop-shop.” This grew out of the business model in the United States in the 1920s and 1930s, with the term used to describe a shop or a company offering multiple services from one location. The model has since been applied to a wide range of areas across different cultural and geographical contexts and has most recently found traction in responses to SGBV. One-stop-shop services offer access to multiple services from a single access point and a single location and have been used as the foundation for progressive DFV service delivery. The Victorian Government's “Orange Door” is one such recent example. Others include the Family Justice Centers in the United States and the United Kingdom. Within this, what has come to be considered best practice is a medical-legal partnership model.

The health-justice or medical-legal partnership originated in the United States in the 1990s and is now globally recognized as a primary model of service delivery to DFV victims. It involves lawyers working alongside health professionals and offers legal services for victims which are colocated in hospitals and community health centers. It is widely known that health professions can play an important role in DFV prevention through, for example, identifying harm and risk, safety planning, and providing referrals to other services (Curran, 2016). Pregnancy and medical illness are often the only points of contact a DFV victim has with a service provider due to the micro-controls she is subjected to within an abusive relationship; these specific opportunities are therefore the only potential help-seeking avenue for some (Gyorki, 2013; Olds, 2006). Health-justice partnerships can help in this regard; however, services can remain highly individualized when they mainly involve creating direct referral pathways from health professionals to on-site legal services, and the model can also sustain the problematic elements of a medicalized approach which we identified earlier. Forell and Gray (2009) caution that, while colocating outreach legal and health services can be a positive endeavour, it is too simplistic to expect that merely colocating these services will in and of itself provide effective assistance to disadvantaged clients.

Reforming the services that respond to DFV is an extremely important part of improving responses and benefiting individual survivors. But our concern is that this does not mean that “systemic change” is under way (see, e.g., RCFV, 2016). We maintain that these changes need to be understood as “systems reform” rather than broad systemic change. A clear distinction needs to be made between responses that seek to improve existing legal and therapeutic response systems, and responses that seek to genuinely address structural inequality—while of course acknowledging that the latter is inherently more challenging. This also entails being alert to the extent to which these health-justice partnerships and other systems reform initiatives can be understood as “holistic” when they can only be understood as holistic to the extent that they attend to multiple immediate individual victim-survivor needs. In the next section, we use the example of CLCs in Australia to differentiate between standard and CLC partnerships. While both can be considered “individual holistic” in that they attend to the multiple needs of victim-survivors, we identify CLC partnerships as unique and progressive because they are bottom-up developed, are grounded in the communities they work in, and give a stronger voice to women's individual and collective intersectional needs.

### Partnering for Structural Change: The Work of CLCs

The CLC approach to DFV is based on a broad, multifaceted, and structurally grounded understanding of the problem. That is, DFV is not a singular physical assault or violent event; rather, it involves a range of pervasive and less visible forms of abuse, and it occurs within the framework of the structural subordination of women.

CLCs have developed a range of partnerships which include health-justice partnerships, court-based and outreach partnerships, finance-justice partnerships, and community partnerships. These focus not only on multiple individual victim needs but also on identifying interconnected structures of violence (e.g., sexism and racism) and advocating for broader social change. As outlined in the previous section, what has become international best practice is the health-justice partnership model. Although useful, these partnerships tend to adopt an individualized approach that does not consider nor address the root of inequality. It is thus important to caution against assuming that a partnership model for addressing DFV has inherent value or that it necessarily leads to structural change. With a focus on quantitative outputs rather than qualitative impacts, it is often unclear what success looks like.

Health-justice partnerships can also present challenges relating to professional boundaries, ethical barriers, confidentiality, lawyer-client privilege, duty of care, and regulatory regimes such as mandatory reporting (Keating, 2017). In their evaluation of the Family Relationship Centre Legal Assistance Partnerships program, Moloney et al. (2011) identified that a common challenge for professionals was working in an interdisciplinary manner when there was significant variation in the histories, cultures, roles, and responsibilities of the disciplines themselves. Breaches of client confidentiality arising from health-justice partnership have also been identified as a particular source of concern because they can jeopardize victims’ safety (Bacchus et al., 2010).

One way that CLCs have attempted to avoid such problems is through their reflexive and collaborative approach to partnership work, with a focus on evolving partnership best practice and groundedness in the community. In establishing program frameworks, CLCs privilege the needs and preferences of victims and navigate ethical issues such as confidentiality through cooperative practice. Ethical challenges such as client-lawyer privilege are therefore addressed through the process of the partnership itself (see Curran, 2016). In giving agency to women's knowledge, ethical issues become not risks to be addressed but rather principles which underpin program structures and victim-survivor engagement more broadly. This runs counter to the “top-down” health-justice partnership model whereby lawyers advise doctors and other health professionals what to look out for when treating possible victims of DFV or when lawyers may advise a specific victim of her immediate legal options. Rather, in being generated “bottom up” through women's collective experiences and through individuals’ experiential expertise, CLC approaches align with critical feminist and other frameworks that argue for lived realities to determine courses of action.

An example that reflects this are programs that tackle women's socio-economic structural disadvantage through targeted anti-economic abuse and women's economic empowerment initiatives. Women's Legal Service Victoria (2018) outlines economic abuse as a form of DFV that often occurs in conjunction with other forms of violence. Economic abuse is a prime example of how DFV negatively impacts a person in multiple, pervasive, long-term ways, and undermines their efforts to become independent (WLSV, 2018). DFV in the form of economic abuse may raise legal and financial issues relating to housing, utility and other bills, debts, fines, and infringements. Financial abuse is often the root cause of poverty experienced by women within and beyond abusive relationships (Branigan, 2007) which confirms the structural socioeconomic roots of DFV.

DFV also remains the “number one cause of homelessness” (Victorian Council to Homeless Persons, 2015, p. 1), and the threat of poverty and homelessness are significant determining factors in victims’ decision-making around remaining in a violent relationship (Branigan, 2007; Cameron, 2014). According to a report of Specialist Homelessness services, 25,104 people (of whom 22,231 were women) who sought assistance from specialist homelessness services in Victoria in 2013/2014 cited DFV as the main problem they faced. Women and children are at increased risk of homelessness because they are often either compelled to leave the home due to violence or because they remain but with significantly reduced household income after the abusive party has left. The Women's Homelessness Prevention Project by Justice Connect (2014–ongoing) keeps women and children in housing through a combination of specialist tenancy legal representation and social work support. It is considered best practice for successfully preventing and reducing housing insecurity. The Restoring Financial Justice program by WEstJustice (2014–2021) also resulted in the adoption of an effective model of economic abuse casework support and revised protocols adopted by industry and community sectors.

This bottom-up approach is also evident in a health-justice partnership in metropolitan Victoria run by the Eastern CLC called Mother and Babies Engaging and Living Safely (MABELS), which provides preventative DFV legal and other support services within the maternal and child health sector. MABELS extends the scope of a common health-justice partnership beyond the dual disciplines of health and law by including DFV victim advocacy and Aboriginal health and healing (Keating, 2017, p. 35). It is an enhanced Maternal Child Health (MCH) program for families where there is an elevated risk of DFV and where there are multiple risk factors. MABELS places a community lawyer and a victim advocate in a number of MCH centers to provide free legal advice, safety planning, information, referrals and community legal education during the same maternal and child health appointment. The project also addresses specific populations in the MABELS catchment: regional, rural, and remote communities as well as Aboriginal and Torres Strait Islander communities. By targeting the most marginalized individuals and groups and by being bottom-up developed, CLC partnerships center women's lived realities. While these programs may not directly lead to structural change and justice, they do provide fertile ground for initiating structural change, as we explore in the next section.

## The Challenge of a Structural Approach to DFV/SGBV

Part of the challenge of pursuing structural change is that it is an encompassing and elusive concept. The concept of structural violence was coined by Johan Galtung (1969, p. 171) who explained it as “indirect” violence which is “built into the structure and shows up as unequal power and consequently as unequal life chances.” Like Galtung, political theorist Iris Marion Young (2006, p. 116) conceptualizes structural injustices as not readily attributable to identifiable perpetrators but rather as a “kind of moral wrong” that is deeply embedded in society's structures and often becomes naturalized and reproduced over time. Structural injustices are manifested in entrenched inequalities which affect particular groups, and which are particularly hard to make visible and address (see also Balint et al., 2020).

To illustrate the intersecting structures of oppression, it is worth looking at the poverty/DFV nexus. Even though economic abuse mostly affects socio-economically disadvantaged women and women of color, it is not only particularly vulnerable women who are at risk of poverty from DFV. In reality, the structural vulnerability of women in society means that all women victims of DFV are at risk of poverty. This is because women continue to be disadvantaged in a multitude of ways that contribute to their economic subordination and their economic vulnerability, most notably within and in the aftermath of an abusive relationship. Understanding DFV as a structural harm means recognizing that it is not just women as individuals but rather women as members of a structurally disadvantaged group that occupy a precarious and vulnerable position. This can be understood through Butler's (2009, p. 25) lens of “precarity”: “a politically induced condition in which certain populations suffer from failing social and economic networks of support and become differentially exposed to injury, violence, and death.”

A structural change approach to DFV prevention also means attending to the *substantive* dimensions of collective structural inequality*.* Women's persistent socioeconomic inequality is perpetuated through systems of employment and social services like Centrelink, the gendered division of labor, childcare, barriers to advancement in career, and intersecting gender and economics through neo-liberal economic shifts such as the casualization of the workforce, which has well-documented gendered impacts (Goodmark, 2018). Addressing DFV, along with other violence against women, requires multiple practical initiatives and programs that aim to address economic inequality such as inequalities in income, employment opportunities and conditions, domestic and other unpaid work, insecure work, and childcare burdens and costs (PHAA, 2015).

Considering the complexity and “politically induced condition” of structural harm, it is understandable that structural change is rarely the main aim of frontline practitioners, who generally need to focus energies and resources on immediate and present problems; they cannot be expected to tackle underlying structural problems since this demands long-term sustained measures. However, in a conversation between three women working to pursue social and legal justice and structural change, legal and advocacy practitioner Meghan Fitzgerald from Fitzroy Legal Service—although cautious about the term “structural change”—provides practical, lived insights into how this is pursued through her work (in Fitzgerald et al., 2016, p. 356):

…structural change is a big, big phrase. I don’t know that I’ve ever approached structural change, but … I work in an organisation that houses the value statements: quality, participation, integrity, respect, empowerment. That's been a very helpful framework for me to work within. … The work that I do is completely dependent on relationships of trust … I work very hard to create lateral, flat structures of power. I’m a feminist. A hardcore feminist. And also I come from the community that I work with. So that's a very important thing. The way I think about my duty is that I need to be a bridge from this place to that place. … And it's a very long stretch but I feel enormously privileged to work with the community that I work with and that community changes all the time. But I tend to work for the people most excluded from discourse because I just need to focus on one thing.

Fitzgerald highlights key elements within the work of CLCs that enable structural change: shared values, creating relationships of trust and flat structures of power, a feminist framework, embeddedness within the community, functioning as a bridge (advocacy/lobbying) and standing up for marginalized people (activism). In the context of Australia, CLCs have attempted to address the structural factors underpinning DFV through a robust history of social activism and their advocacy and lobbying work is crucial to changing dominant, reductive understandings of DFV, fostering networks with communities at risk of DFV and addressing multiple vulnerabilities and systems of oppression. Although the term structural change is rarely mentioned in official CLC documents, they have been at the forefront of broader advocacy efforts for disadvantaged groups and are well recognized for their more radical lobbying for social equality and system-wide structural change (McCulloch & Blair, 2012).

Some critics argue that CLCs are now firmly embedded in state delivery structures and have thus lost their ability to critique and reform those structures. Rice (2012), for example, argues that, in order to maintain their radical reformist roots, CLCs need to remain in contestation with the state rather than functioning as outreach service providers for the government. On the other hand, Noble (2012, p. 23) points out that “the potential of CLCs to reach beyond direct service delivery and engage in proactive advocacy and policy work is […] their greatest strength”. To challenge the structures that produce inequalities and hierarchies is essential to combat DFV. CLCs therefore must keep close to their radical activist roots and continue to reflect on their role and purpose. This is what community lawyer Fitzgerald refers to as “civil disobedience” and where active community-based legal education and communication can enable people to engage creatively with the law and contribute to societal change. This is especially important because litigation as a “critical tool in holding the state to account […] doesn’t always work that well” (Fitzgerald et al., 2016, p. 362).

Remaining close to their radical roots is particularly important for CLCs in light of activist academic work which emphasizes the need to challenge how DFV is conceptualized and approached. This is because, despite DFV being acknowledged as a structural problem in policy reports and feminist scholarship, it is rarely understood or responded to accordingly. Goodmark (2018), for example, critiques the dominant conceptualization of DFV as a crime problem to be tackled through local criminal legal systems and increasingly punitive responses; instead suggesting it be approached as an economic, health, and international legal and political problem. Rose (2015, 2022) goes further and argues for the reconceptualization of DFV as a state crime, highlighting that DFV is produced not just by violent individuals but by the very states, structures, and institutions that claim to be combating the problem, and that prevention is therefore a much broader project. This work links with other scholarship that highlights the need to understand mass harms not as a series of singular and discrete acts but as broader institutional and structural harms which are an outgrowth of their historical and cultural context (e.g., Balint, 2012, 2016). Understanding DFV as structural violence means moving beyond an individual perpetrator model to consider how states, institutions, and their policies and laws through everyday practices (re)produce sexual and gendered inequalities. It means moving toward substantive and genuinely structural reform which changes not just the material realities of individual victims nor the harmful attitudes of individuals or groups but which targets whole institutions, structures, and societies. This requires a critical yet collaborative relationship with the state.

Addressing DFV structurally also necessitates that prevention remains connected to local and global feminist and other movements for structural and social justice. These include abolitionism, decolonization, environmentalism, and other movements which practise and promote nonviolent, needs-based, and community-based organizing, activism, and interventions in the place of privilege-based organizational, institutional, and state harm. In such movements, people have gathered to challenge white supremacy, patriarchal power, rampant capitalism, corporate crime, and carceral logic, aiming to create safety from harm and especially from the harms of the powerful.

In their work, CLCs intuitively align their work with intersectional feminist and decolonial theory that illuminates interrelated systems of oppression and shows how SGBV is understood within a continuum of violence (Crenshaw, 1989; Kelly, 1988; Scheper-Hughes & Bourgois, 2004; Verges, 2020). Kelly (1988) conceptualizes this continuum as a spectrum of victimization experiences that pervade the everyday life of women and girls within an overarching sexual and gendered order of inequality and domination. Intersectional feminists have added the necessity of race-based analyses within this also. Crenshaw (1989), for instance, argues that white women's critiques of patriarchy often fail to consider the experiences of Black women who face race-based oppression, and which cannot be addressed separately. Gender-based violence exists along a continuum of violence as it is immersed in racism, classism, sexism, colonialism, poverty, and other forms of structural violence. Accordingly, responses to DFV and other SGBV need to factor in the intersecting structures of oppression as well as specific women's experiences, thereby linking the structural with the individual experiential. CLCs’ specialized work with victims of DFV through their embeddedness in local communities affords them an in-depth understanding of the priorities and needs of specific locales and groups, enabling them to adopt both structurally grounded and targeted approaches that address these unique needs and also communicate these to policymakers.

It is here that CLCs and other similar grassroots organizations form a crucial bridge between the specific needs of communities and the bigger power structures that produce and reinforce inequalities. This approach, guided by an ethics of partnership, can identify the structures that require change. Through the solutions proposed that emerge from the embeddedness of CLCs *within* the community, these are collaborative solutions that meet community needs and understand how the structural barriers are experienced on the ground and in the everyday. A partnership of practice that identifies structure is, at its heart, collaborative and activist work: it is dependent on who you are partnering with and whose voices need to be heard. It is through such a partnership that structures of harm can be identified because being survivor-centered illuminates structures that are most often invisible. This active approach to partnership is quite different from simply a colocation or service-integration approach. The CLC model is one in which partnerships are generated from the ground up and are community and survivor-centered. They thus help identify the broader structural patterns and problems that need to be addressed.

This also means listening attentively to survivors’ experiences and insights about DFV and other SGBV response and prevention. During research in the eastern Congo by one of the authors, women survivors of sexual violence emphasized the importance of providing education, food, and security for their children and other family (Mertens & Pardy, 2017). Crucially, these needs were considered far more important than having the harm caused by their victimization acknowledged or seeing perpetrators punished. This highlights how victims themselves—even when immersed in their own personal trauma recovery process—possess remarkable insights about what is most needed to counter the reproduction of victimization experiences like their own. Indeed, these survivors saw the need to eradicate the structural violence of war, insecurity, poverty, and hunger as *more urgent* than legal redress or retribution. Incorporating insights like these is essential in supporting shifts beyond punitive justice and pursuing more meaningful change in society.

What thus discerns the work of CLCs from the more top-down oriented integrated partnership model is its embeddedness in community, which means that communities themselves often own and manage structures and processes. This approach to partnerships goes beyond the colocation individual service model which does not acknowledge that everyone has their own unique experiences of discrimination and often overlooks intersecting structures of oppression. As noted by Crenshaw (1989, p. 167), “the failure to embrace the complexities of compoundedness is not simply a matter of political will, but is also due to the influence of a way of thinking about discrimination which structures politics so that struggles are categorized as singular issues.” In this sense, she argues, it is of the utmost importance to start by addressing the needs and problems of the most intersectionally disadvantaged people. This “plac[es] those who currently are marginalized in the center [and] is the most effective way to resist efforts to compartmentalize experiences and undermine potential collective action … When they enter, we all enter” (Crenshaw, 1989, p. 167; also see Gangoli et al., 2019; McGlynn & Westmarland, 2019).

The intersectional partnership approach is exemplified by the Marninwarntikura Women's Resource Centre in Fitzroy Crossing in Western Australia (WA), part of the Violence Prevention Legal Services. This center's name draws from the local Indigenous language in which “Marnin” means “women,” “Wanti” means “big mobs of women,” and “Kura” means “belonging to.” Located in rural Australia, this service offers diverse support to Aboriginal and Torres Strait Islander women and children who have experienced DFV. The service is colocated with a women's refuge and offers support through counseling and legal advice but also offers programs that empower women economically, culturally, and politically. It is built on the premise that Indigenous peoples are best placed to identify the challenges they face, and also the solutions. This is reflected in the service's Board which is directed by local Indigenous women from each of the main language groups. For Indigenous peoples, an integrated response does not mean colocation (e.g., having a doctor and lawyer at the same site); rather, it means a focus on specific violence prevention together with cultural health and healing families (Blagg et al., 2018). Cultural strength and identity as well as country-centered approaches are key to addressing the intergenerational trauma of colonialism with its dispossession and destruction of cultural practices. This service recognizes that certain approaches considered “best practice” in a Western context are poorly suited to the needs of Indigenous women. For example, failing to recognize how, for Indigenous women, leaving an abusive partner can go against “cultural and family obligations of Indigenous women, which makes ‘exiting’ family relations particularly difficult” (Blagg et al., 2018, p. 53), can result in the problematic and even harmful imposition of so-called “best practice” initiatives like Family Violence Intervention Orders.^3^

While Indigenous women are particularly at risk of partner and other family and kinship violence, it is crucial to understand this violence in the context of a history of racism, colonization, land dispossession, forced child removal, and resulting intergenerational trauma (Blagg et al., 2018; Cunneen & Tauri, 2016). DFV is an outcome of patriarchal, colonial, and capitalist gender orders. As shown by feminist decolonial thinkers (Lugones, 2007; Verges, 2020), the colonial power matrix of capitalism and a particular racialized, sexualized, and classed gender order promotes and naturalizes domination by economically privileged white men and exploitation of certain groups of people, notably Black women and women of color. In Australian and other colonial contexts, a European “dual sex/gender system” tended to eclipse Indigenous understandings of gender which were generally considered much more fluid and flexible (Tamale, 2020, p. 100), while sexual violence was central to the execution of colonial power and the survival of the colonial nation-state (Mertens, 2023). Today, the foundational racialized inequalities of empire endure through the continuing oppression and violation of all women's bodies, and for Indigenous women in the settler-colonial context of Australia, this has taken specific forms such as genocide (Blagg et al., 2018; Lucashenko, 1996; Porter & Cunneen, 2020; Watson, 2007). It is in the identification of structural violence and the interconnected factors that produce it—in partnership with communities—that these kinds of services facilitate illuminating and addressing DFV as a structural phenomenon.

Structural change initiatives to address DFV then need to address the “interlocking nature of oppression” (Collins, 1986, p. 19) which can only be done through a multi-pronged intersectional feminist ethics and praxis which links the structural to the experiential. It is in CLCs’ relative independence from the government and their embeddedness within those communities that structural change is possible. As articulated by Fitzgerald: “I’m fighting and trying to create community as well … sometimes people can create structural change by working together and by just believing in what they’re doing. It just happens” (Fitzgerald et al., 2016, p. 362). This reminds us of how we must be working together to create sustainable and structural change.

## Conclusion

As critical anti-violence scholars, we consider it our role to ask why and how we are addressing harms in certain ways and not others, and to initiate and drive broader—and inherently more challenging—conversations around what needs to happen to address structural inequality, not merely its symptoms. We argue that *systemic change* has—or should have—an entirely different meaning than updating DFV response and service systems; it should reflect an understanding of the complex and inter-connected political, social, cultural, and institutional histories, systems, and structures that constitute our local and global societies and which are responsible for producing structural problems like sex-, race-, and class-based inequality, and of course DFV and other forms of SGBV.

While there is undeniable value in targeted anti-DFV measures like those discussed early in this article, we need to be critically aware of how they can obscure more accurate conversations about what DFV and other SGBV is, and what its prevention demands. Even progressive survivor-centered anti-violence work generally maintains an individualized understanding of the problem and is limited to immediate—and sometimes helpfully integrated—medical or legal remedies for victim needs. Positive perpetrator-focused initiatives that promote accountability or behavior change, for example, are also individual in focus and approach. Yet, all SGBV is a problem of structural violence as well as interpersonal violence, and without addressing this, approaches will have a limited preventative impact. It is therefore vital, amid more targeted prevention efforts, to keep sight of the historical, institutional, and structural roots of DFV and other SGBV and the consequent need for measures that target these dimensions of the problem.

We looked at the partnership approach pioneered by CLCs, which has had success in treating the multiple dimensions of DFV through multifaceted survivor needs that also relate to structural disadvantages. Yet a survivor-centered partnership approach does not always mean an approach is structural; however, any structural approach is necessarily survivor-centered. What is distinct about a structural approach to DFV redress and prevention is that it emerges from communities, is colocated in communities, and is activist in nature. This provides a way of identifying structures of violence and how these might be addressed. We also reflected on the approach taken by CLCs to consider how they use partnerships to work toward structural change. They are located where they need to be: in maternity child health centers, in libraries, on the street, and in remote communities; and they can be adaptive and responsive. They are not solely micro-solution-driven but are focused on the needs of the communities they work in and with. They adopt a feminist and decolonial framework, can function as a bridge between vulnerable groups and policymakers, and continue to stand up for the most marginalized. This work requires a certain degree of “civil disobedience”—a willingness to work within, outside, and against the law to imagine and achieve structural change informed by and activated at the grassroots level.

## Notes

1. In this article, we use the term DFV to refer to a broad spectrum of coercive, controlling, abusive or violent behaviours perpetrated by men against female intimate partners or ex-partners. Whilst acknowledging that DFV can refer to other harms including child, elder, or carer abuse, as well as violence involving broader kinship and community networks (see Blagg et al., 2018; Cunneen & Tauri, 2016), our focus here reflects local and global knowledge that male-on-female intimate partner violence is the most prevalent and harmful form of not just DFV, but of all sexual and gender-based violence.

2. These shifts remain an area of contestation, with ongoing questions remaining as to whether specifically criminal legal changes can or do in reality enhance the recognition of or response to DFV victim-survivors (see Walklate et al., 2019).

3. These are also known as Domestic Violence Orders, or Restraining Orders, in some contexts.
